# Identification of individuals at high-risk for pancreatic cancer using a digital patient-input tool combining family cancer history screening and new-onset diabetes

**DOI:** 10.1016/j.pmedr.2023.102110

**Published:** 2023-01-16

**Authors:** Derk C.F. Klatte, Kristin E. Clift, Sarah K. Mantia, Lindsey Millares, Sanne A.M. Hoogenboom, Richard J. Presutti, Michael B. Wallace

**Affiliations:** a Department of Gastroenterology and Hepatology, Mayo Clinic, Jacksonville, FL, USA; b Department of Gastroenterology and Hepatology, Leiden University Medical Center, Leiden, The Netherlands; c Department of Clinical Genomics, Mayo Clinic, Jacksonville, FL, USA; d University of Florida, Gainesville, FL, USA; e Department of Family Medicine, Mayo Clinic, Jacksonville, FL, USA; f Department of Gastroenterology, Sheikh Shakhbout Medical City, Abu Dhabi, United Arab Emirates

**Keywords:** Family history, Genetic counseling, Genetic testing, Hereditary pancreatic cancer

## Abstract

Capturing family history might be a valuable tool for identification of individuals at increased risk of pancreatic cancer, which would allow enrollment into pancreatic surveillance programs. In addition, weight loss and concurrent new-onset diabetes may be utilized as an early marker for pancreatic cancer. This study evaluates the yield of combining family history and the Enriching New-Onset Diabetes for Pancreatic Cancer (ENDPAC) model to identify individuals who could benefit from pancreatic surveillance. A novel questionnaire and digital input tool was created that combined questions on family cancer history and criteria of the ENDPAC model. Individuals meeting ENDPAC criteria were enrolled directly in the high-risk pancreatic clinic. Individuals who met the criteria for a significant family history of cancer were offered referral to a genetic counselor. The questionnaire was completed by 453 patients. Of those, 25.8% (117/453) had significant familial risk factors. Eighteen individuals (15.4%) completed genetic testing previously, of whom five had a pathogenic variant. Thirty-four (29.9%) out of 117 individuals with a strong family history – flagged by the questionnaire – underwent genetic testing. Four (11.8%) of these patients harbored a pathogenic variant. Additionally, through cascade family testing, two siblings were found to carry pathogenic variants. Four (0.9%) of the 453 patients matched ENDPAC criteria. Two were diagnosed with pancreatic cancer and the others were enrolled in the surveillance program. In conclusion, identification of high-risk individuals for pancreatic cancer can be achieved by combining family history screening and the ENDPAC model to facilitate referral to genetic counseling and high-risk clinics.

## 1 Introduction

The United States Preventive Task Force reaffirmed in 2019 that asymptomatic, population-based screening for pancreatic cancer is not recommended (Owens et al., 2019). The reason for this is that the average lifetime risk is too low and would therefore most likely result in a large number of false-positive findings. However, incidence of pancreatic cancer is on the rise and will soon become the second leading cause of cancer death in the United States (Pourshams et al., 2019). It is therefore ever more important to identify individuals at high risk for pancreatic cancer who could benefit from surveillance.

It is estimated that up to 10 % of pancreatic cancer cases arise in individuals with a strong family history or in carriers of a germline mutation (Whitcomb et al., 2015), for which pancreatic surveillance is recommended (Goggins et al., 2020). Recent evaluations of surveillance programs suggest that individuals who might benefit from surveillance are likely to be restricted to the ones carrying a pathogenic variant in a cancer predisposition gene (Overbeek et al., 2021, Klatte et al., 2022a). Stratification of high-risk individuals would ensure that surveillance is offered to those who could benefit the most, while minimizing potential harms of screening (Klatte et al., 2022b). Capturing family cancer history offers a simple and cost-effective way to identify individuals at increased risk for pancreatic cancer and hereditary cancers in general (Lucas et al., 2017, Valdez et al., 2010, Wang et al., 2007). Darabi et al. (Darabi et al., 2020) demonstrated that in a community setting, involving patients with a personal and/or family history of cancer (n = 8,239), genetic counseling and subsequent germline mutation testing resulted in identification of pathogenic or likely pathogenic variants in 15 %. This underlines the significance of genetic counseling and testing to identify those who could potentially benefit from pancreatic surveillance (Klatte et al., 2022a, Dbouk et al., 2022). Both the American Society of Clinical Oncology (ASCO) and National Comprehensive Cancer Network (NCCN) recommend that patients diagnosed with pancreatic cancer should undergo assessment of risk and subsequent genetic testing in case of a suspected hereditary cancer syndrome (Stoffel et al., 2019, Tempero et al., 2021). In addition, germline genetic testing may also be offered to pancreatic cancer patients (or their first degree relatives) even when family history is unremarkable (Stoffel et al., 2019).

A three-generation pedigree is the gold standard for appraising risk of an autosomal dominant cancer syndrome, of which family history of cancer in first- and second-degree relatives is most relevant. However, several barriers exist to family history collection in the clinical setting. Self-reported histories can be limited, inaccurate or static (Lu et al., 2014). Moreover, providers are often restricted in time, are overwhelmed with data and “alert fatigue” (Ancker et al., 2017, Hamilton et al., 2017), and there are gaps in knowledge about hereditary cancers and genetic testing (Hamilton et al., 2017, Dekanek et al., 2020). These factors make it challenging to reliably quantify hereditary risks. In consequence, patients with a genetic predisposition for cancer may be missed and thereby not receive counseling on individualized cancer surveillance.

In addition to a genetic predisposition, individuals with new-onset hyperglycemia and diabetes (NOD) have an increased likelihood (approximately 8 fold higher risk) of developing pancreatic cancer, compared to the general population (Pannala et al., 2009). As such, recognition of NOD as an early sign of a pancreatic malignancy could aid in the diagnosis of early stage cancers (Chari et al., 2008). However, a major challenge amidst the epidemic of obesity is to distinguish this pancreatogenic (type 3c) diabetes from type 2 diabetes. Sharma et al. (Sharma et al., 2018) developed and validated a model based on change in weight, change in blood glucose, and age at diagnosis of diabetes. The resulting Enriching New-Onset Diabetes for Pancreatic Cancer (ENDPAC) model identified patients who developed pancreatic cancer within 3 years of diabetes onset (area under receiver operating characteristic curve 0.87). Thus, identifying patients with NOD could be an additional potential strategy to further enrich our high-risk cohorts with individuals who could benefit from relatively short-term (≤3 years) participation in pancreatic cancer surveillance.

In this pilot study, we evaluated combining family history and assessment of NOD in a gastroenterology and hepatology outpatient clinic to identify individuals at high-risk of pancreatic cancer who could benefit from genetic testing, and subsequent enrollment in pancreatic surveillance.

## 2 Methods

### 2.1 Setting and study population

This project was conducted at the Department of Gastroenterology and Hepatology of Mayo Clinic Florida, United States. The study population included patients ≥ 18 years seen at the outpatient clinic for any gastroenterology (GI) indication between August 2018 and May 2019.

### 2.2 Pancreatic Cancer Risk Tool questionnaire

The Pancreatic Cancer Risk Tool (PCRT) questionnaire is an application (app)-based questionnaire that was created in collaboration with Input Health (InputHealth, Ontario, Canada: a company specializing in patient-input health tools). The PCRT included questions on family history, weight change and fasting blood glucose change. This included questions on personal history of cancers (e.g. *“Have you ever had one of the following cancers?”)* and family history of cancers *(*e.g. *“What is your biological mother’s cancer history?”; “Was your mother diagnosed with any of the above cancers before or at the age of 50?”)*. The complete questionnaire is provided in Supplemental Table 1.

The family history questions were backed with a simple scoring algorithm based on the NCCN 2020 guideline to prompt referral for genetic counseling (Daly et al., 2020). A significant familial risk score was defined as having a score greater than or equal to three; which was an indication for referral to genetic counseling. Example 1: Personal or Family history of pancreatic cancer = 3 points. Example 2: Personal or family history of breast cancer = 1 point, if under the age of 50 add 2 points. Because several genes and cancer syndromes are associated with a high pancreatic cancer risk, questions were asked about family cancer history in general. Patients who proceeded with genetic testing were tested with a multi-gene hereditary cancer panel. Tests varied based on patient’s personal history, family history, personal preferences, and insurance.

The questionnaire asked patients about recent weight changes and recent fasting blood glucose changes in attempt to identify people meeting criteria from the ENDPAC model. Additionally, questions were included on other risk factors such as smoking, pancreatitis, obesity, and duration of diabetes.

The questionnaire was e-mailed to all patients visiting the outpatient GI clinic in advance. If patients had not filled in the questionnaire in advance, the questionnaire was offered on a tablet device in the waiting room. Prior to filling in the questionnaire, patients were offered education about the purpose of the questionnaire, namely the identification of people with a higher than average risk of developing pancreatic cancer or other cancers (as part of hereditary cancer syndromes). Patients with significant risk factors identified (high-risk), were contacted by their GI provider and offered referral to a clinical geneticist for further evaluation.

This study complies to Mayo Clinic’s guidelines for protection of human subjects safety and privacy. Deployment of the PCRT questionnaire was permitted as part of a quality improvement study. Permission to conduct retrospective chart review to capture additional clinical data was given by the Mayo Clinic Institutional Review Board (ID: 19–010097).

### 2.3 Statistical analysis

For descriptive analyses, continuous variables are reported as a mean with standard deviation (SD) or median with interquartile range (IQR). Categorical variables are reported as frequencies with percentage of total. All statistical analysis were performed using R version 4.0.2.

## 3 Results

**Fig. 1:**
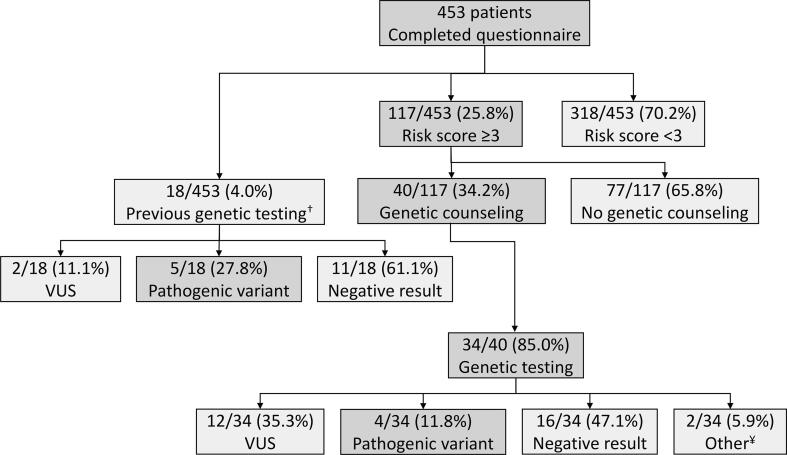
Overview of outcomes of patients who completed the Pancreatic Cancer Risk Tool questionnaire VUS = variant of uncertain significance ^†^ Three out of 18 individuals who underwent previous genetic testing had a risk score < 3 ^¥^ One patient was found to be a carrier of Bloom syndrome (BLM gene) and one patient was found to be a carrier for Gilbert syndrome (UGTA1 gene).

The questionnaire was completed by 453 patients who visited the Gastroenterology and Hepatology clinic between August 2018 and May 2019 (Fig. 1). Since all visiting patients were e-mailed the questionnaire in advance and were offered the questionnaire again in case the questionnaire was not filled in, we expect the completion rate to be nearly 100 %. A little over half of the participants were female (251; 55.4 %), with a median age of 65 years (IQR 54 – 72). Six of the 453 patients (1.3 %) had a pre-existing diagnosis of pancreatic cancer, and 88 out of 453 individuals (19.4 %) reported having a personal history of one or more different cancers. The median risk score was one (IQR 0 – 3) and the highest score was 13. In total, a quarter (117/453; 25.8 %) of patients were identified to have significant familial risk factors (risk score ≥ 3). Eighteen patients (18/453; 4.0 %) had previously undergone genetic testing prior to completing the questionnaire. Three of these 18 patients had a risk score lower than three. Demographic data for respectively the low-risk and high-risk group are summarized in Table 1.

**Table 1:** Demographic information for 453 respondents to the Pancreatic Cancer Risk Tool questionnaire (PCRT).

	PCRT < 3 (n = 318)	PCRT ≥ 3 (n = 117)	*P-value*
Female, n (%)	170 (53.5)	67 (57.3)	0.48^
Age, median (IQR)	64 (51.2 – 72.0)	66 (60.0 – 73.0)	0.02#
Ethnicity, n (%)			0.39†
	White	286 (89.9)	108 (92.3)
	African American	23 (7.2)	4 (3.4)
	Asian	4 (1.3)	2 (1.7)
	Other	5 (1.6)	3 (2.6)
Diabetes or glucose intolerance, n (%)	72 (22.6)	32 (27.4)	0.37^
Currently tobacco smoking, n (%)	23 (7.2)	10 (8.5)	0.80^
Personal history of any cancer, n (%)	46 (14.5)	38 (32.5)	<0.01^
Ashkenazi Jewish ancestry*, n (%)	7 (2.2)	16 (13.7)	<0.01^

IQR = interquartile range.

* Biological mother, father or both.

^ Chi-square test.

# Mann-Whitney.

† Fisher’s exact test.

All patients with familial risk factors (risk score ≥ 3) were informed of the high-risk clinic and offered consultation with a genetic counselor. Due to the broad nature of the cancer family history questions asked, the questionnaire identified people at risk for hereditary cancer syndromes in general. Thirty-four individuals who were flagged by the questionnaire, completed genetic testing for the first time. Of those, four individuals (4/34; 11.4 %) were found to harbor pathogenic variants (Table 2). Five (5/18; 28 %) of the 18 individuals who previously underwent genetic testing, harbored a pathogenic variant (Fig. 1; Supplemental Table 2). Thus, including the previously identified patients, nine (18.4 %) out of the 49 patients who had a risk score ≥ 3 and underwent genetic testing had a pathogenic variant.

**Table 2:** Characteristics of patients identified with a pathogenic variant from the Pancreatic Cancer Risk Tool questionnaire.

Age/sex, reason of visit	Gene	Variant	Phenotype	Familial risk score, personal and family history
54/F Abdominal pain	*RET*	c.1826G > A (p.Cys609Tyr)	Multiple endocrine neoplasia type 2	Score 8: personal history of thyroid cancer; sibling with breast cancer under 50; mother with ovarian cancer under 50
65/F Pancreatitis	*ATM*	c.7630 −2A > C (splice acceptor)	Hereditary susceptibility to cancer	Score 6: Ashkenazi Jewish mother with pancreatic cancer; father with prostate cancer
75/M Colon cancer screening	*APC*	c.3920 T > A (p.Ile1307Lys)	Low penetrance colorectal cancer risk allele	Score 6: both parents are of Ashkenazi Jewish decent and mother has a history of breast and colon cancer under the age of 50
67/F Malignant neoplasm in pancreatic head	*SDHA*	c.91C > T (p.Arg31X)	Hereditary susceptibility to paraganglioma	Score 6: personal history of pancreatic cancer and mother was diagnosed with breast cancer under the age of 50

Of the four patients identified in this pilot with pathogenic variants, one patient was found to have a low penetrance *APC* risk allele for colorectal cancer. One patient was molecularly confirmed to have multiple endocrine neoplasia type 2 due to a pathogenic variant in the *RET* gene, which allowed for cascade screening in her sibling who also carried the variant. Another patient was found to carry an *SDHA* variant associated with risk for paraganglioma and pheochromocytomas. Lastly, a patient with a recent episode of pancreatitis was diagnosed with pancreatic cancer after she filled out the questionnaire and was found to carry a pathogenic *ATM* variant. The result prompted consideration of platinum-based chemotherapy and/or PARP inhibitors in her cancer management. Her unaffected sibling also tested positive for the familial variant and has implemented a new cancer surveillance regimen.

Sixteen (47.1 %) of the 34 patients received negative results, and twelve (34.4 %) patients harbored one or more variants of uncertain significance (VUS). There were two secondary findings: one patient was found to be a carrier of Bloom syndrome (*BLM* gene), and one patient was found to be a carrier for Gilbert syndrome (*UGTA1* gene).

Questions assessing NOD criteria adopted from the ENDPAC model were completed by 348 (76.8 %) out of 453 patients. Of those patients, approximately-one-third (128/348; 36.8 %) did not know whether any changes in fasting blood glucose occurred and 16 (4.6 %) could not recall their weight one year ago. Thus, 220 (63.2 %) out of 348 patients were able to provide sufficient information to be assessed for the NOD criteria. Four patients (4/220; 1.8 %) had self-reported weight-loss and self-reported higher than normal blood glucose around the age of 50 who were not yet diagnosed with pancreatic cancer (GI appointment indications: diarrhea and flushing; pancreatic cyst; pancreatitis; pancreatic mass). Other risk factors were reviewed, and these patients were offered to be enrolled in the high-risk clinic. One patient is currently being monitored with annual magnetic resonance imaging (MRI). Two patients were diagnosed with pancreatic cancer shortly after filling out the questionnaire (Table 3). The first patient was a 59 year old female who came for evaluation of a small pancreatic cyst (6 mm) with mild pancreatic duct dilation in the tail without any suspicion of a pancreatic mass on computed tomography (CT). Within the last year she lost 14 % of her bodyweight, which she said was intentional. She had no other (abdominal) complaints. Repeat pancreatic imaging with MRI was performed nine months later, which showed an increase of duct dilation and suggestion of a pancreatic mass, which was confirmed on endoscopic ultrasound (EUS). Patient underwent a distal pancreatectomy and was diagnosed with pT1cN0M0 pancreatic cancer. The second patient was a 81 year old female who came for evaluation of a pancreatic mass, which was found as an incidental finding on CT to rule out kidney stones. She reported a 4 % loss of bodyweight. Imaging showed a 16 mm pancreatic mass with upstream dilation of the pancreatic duct and atrophy. Patient underwent neoadjuvant chemoradiotherapy because of arterial and venous encasement. Seven months later, a distal pancreatectomy was performed an patient was diagnosed with ypT1apN0M0 pancreatic cancer with positive resection margins, and underwent subsequent adjuvant chemotherapy.

**Table 3:** Characteristics of patients who met Enriching New-Onset Diabetes for Pancreatic Cancer (ENDPAC) criteria and were later diagnosed with pancreatic cancer.

Age/sex, reason of visit	Self-reported current and previous weight	Timeline from questionnaire completion to diagnosis and treatment	Familial risk referral score
59/F Pancreatic cyst	Current weight 209 lbsPrevious weight 242 lbs (-14 %)	• January 2019 – CT: 6 mm pancreatic cyst with mild pancreatic duct dilation; no suspicion of mass • March 2019 – Questionnaire completed • October 2019 – MRI: increase of duct dilation with suggestion of a mass • November 2019 – Distal pancreatectomy. Pathology: 16 mm well differentiated adenocarcinoma arising from intraductal papillary mucinous neoplasm (pT1cN0M0), with negative resection margins • January 2020 – Adjuvant chemotherapy	Score 1: father with colorectal cancer)
81/F Pancreatic mass	Current weight 175 lbsPrevious weight 183 lbs (-4%)	• January 2019 – Questionnaire completed • January 2019 – Diagnosis of pancreatic cancer and start of neoadjuvant chemoradiotherapy • October 2019 – Distal pancreatectomy: ypT1aN0M0 pancreatic cancer, with positive resection margins • November 2019 – Adjuvant chemotherapy	Score 3: sibling with pancreatic cancer Previous genetic testing negative

## 4 Discussion

Our study found that a simple, low-cost, non-invasive application-based questionnaire was effective at identifying a relevant number of individuals at elevated risk for hereditary cancer and met guidelines for further genetic evaluation and/or referral for pancreatic cancer surveillance. Four individuals identified through our questionnaire harbored a pathogenic variant, of which two (*ATM* and *APC*) are considered relevant pancreatic cancer susceptibility genes (Goggins et al., 2020, Klatte et al., 2022a). This renders the eventual yield of the family history questionnaire in this population relatively modest. An additional four patients met ENDPAC criteria, of which two were subsequently diagnosed with pancreatic cancer. This study supports that patient-reported family cancer histories are a critical component of cancer risk assessment, which allows for subsequent genomic evaluation and potential enrollment into surveillance programs (Randall Armel et al., 2009, Rich et al., 2004, Frezzo et al., 2003, Kallenberg et al., 2015).

By offering the questionnaire to patients we were able to achieve an incremental gain for uptake of genetic testing. Only 18 (4 %) out of the 453 patients who completed the questionnaire had previously undergone genetic testing. Following completion of the questionnaire and subsequent genetic counseling, this proportion almost doubled. An additional way to identify patients at risk of pathogenic variants is through cascade screening for asymptomatic family members (Caswell-Jin et al., 2019, Frey et al., 2020). Consequently, this pilot identified two siblings who also carried pathogenic variants. In future studies, a more concentrated effort to offer cascade screening to family members would maximize the value of similar risk identification tools.

Of relevance is that approximately-one-third of the patients who underwent genetic testing were diagnosed with a VUS. Due to sensitive sequencing technologies, gene variants can be detected for which the functional impact and clinical consequence is uncertain (Moghadasi et al., 2016). This can be a challenging concept to comprehend and may cause anxiety, worry and uncertainty in counselees (Richter et al., 2013, O'Neill et al., 2009, Chiang et al., 2021). As genetic testing will likely continue to increase, it is essential to improve education for patients and providers to ensure correct understanding and management (Plon et al., 2011, Makhnoon et al., 2019). Concurrently, further research is required to reclassify a proportion of VUS into clinically actionable variants (Macklin et al., 2018, Dettwyler et al., 2022).

One of the limitations of this study include the known limitations of relying on patient-reported data. Although literature shows that cancer family-history questionnaires are in general reliable (Murff et al., 2004), we observed that regarding the ENDPAC model, many patients could not recall or did not know if they’ve had a rise in blood glucose levels. Because this study relied on patient input data, we identified a low number of patients meeting the ENDPAC model criteria. Factors to identify NOD and weight change are mostly readily available in the electronic medical record, which could be used to automatically assess risk and identify individuals who are at increased risk of pancreatic cancer.

Family history should be reviewed with patients frequently. Unfortunately, family history is often overlooked because of competing demands in current practices and data overload. A potential solution is to incorporate the referral algorithm in the electronic medical record to automate identification of patients who should be referred to genetic counseling. To subsequently further improve uptake of genetic testing, efforts should focus on mitigating some of the associated barriers. In this study, less than one third (34/117; 29.1 %) of the individuals with significant familial risk factors engaged with genetic testing. These findings are in line with a previous study, which showed comparable numbers for referral rates and testing engagement from using a patient-entered method for gathering family history (Buchanan et al., 2015). Some of the reasons noted by patients who did not move forward with genetic testing were lack of interest, concerns about costs, insurance coverage, and timing. These factors could be alleviated in the future with upfront information about actual costs and insurance coverage, information about The Genetic Information Non-Discrimination Act (GINA, 2008), timely coordination and communication of results and referral for genetic counseling.

In conclusion, identification of high-risk individuals for pancreatic cancer can be achieved in a low-risk and low-cost way by combining family history screening and the ENDPAC model criteria in a questionnaire to facilitate referral to genetic counseling and enrollment in high-risk surveillance programs.

## CRediT authorship contribution statement

**Derk C.F. Klatte:** Methodology, Formal analysis, Investigation, Writing – original draft. **Kristin E. Clift:** Conceptualization, Methodology, Investigation, Project administration, Funding acquisition, Writing – review & editing. **Sarah K. Mantia:** Methodology, Data curation. **Lindsey Millares:** Data curation, Writing – review & editing. **Sanne A.M. Hoogenboom:** Validation, Writing – review & editing. **Richard J. Presutti:** Conceptualization, Methodology, Resources, Writing – review & editing, Supervision. **Michael B. Wallace:** Conceptualization, Methodology, Funding acquisition, Investigation, Supervision, Writing – review & editing.

## Declaration of Competing Interest

The authors declare that they have no known competing financial interests or personal relationships that could have appeared to influence the work reported in this paper.

## Data Availability

Data will be made available on request.
