# Supplementary material for: Identification of individuals at high-risk for pancreatic cancer using a digital patient-input tool combining family cancer history screening and new-onset diabetes
Source: Prev Med Rep. 2023 Jan 16;31:102110. doi: 10.1016/j.pmedr.2023.102110 (PMC9938327; doi:10.1016/j.pmedr.2023.102110)
Supplement: Supplementary data 1 [file mmc1.docx]

**Supplemental materials**Identifying high-risk individuals for pancreatic cancer with family history and new-onset diabetes

**Supplemental table 1.** Content of the Pancreatic Cancer Risk Tool questionnaire

| 1. **What is your ethnicity?**  American Indian or Alaska Native  Asian (including Indian subcontinent and Philippines)    Black or African American (including Africa and Caribbean)   Native Hawaiian or Other Pacific Islander   White   Other, please specify | |
| --- | --- |
| 1. **Do you currently smoke cigarettes?**  Yes   Age started smoking   Average number of cigarettes per day  No – Never smoked  No - Quit   Age started smoking   Average number of cigarettes per day   Age quit smoking | |
| 1. **Do you consume any other tobacco products?**  Yes  No | |
| 1. **Have you ever had pancreatitis?**  Yes  No  I don’t know | |
| **ENDPAC MODEL QUESTIONS** | |
| **Have you ever been told you had diabetes or a problem with high blood sugar?**  No  Yes   Type 1 Diabetes   Type 2 Diabetes   Type 3 Diabetes   Unsure | |
| 1. **Have you had diabetes for more than 5 years?**  Yes  No | |
| 1. **Do you know if there has been a change in your fasting blood glucose recently?**  Yes, it was higher than normal  Yes  No  I don’t know | |
| 1. **What is your height?** [enter height] | |
| 1. **What is your weight?** [enter weight] | |
| 1. **Do you recall your weight 1 year ago?**    No, but I had weight gain  No, but I had weight loss  No, but there was no change  No, not at all  Yes {enter weight] | |
| 1. **If you lost weight in the last year, was your weight loss intentional?**  Yes  No    Unsure  I did not lose weight |  |
| **FAMILY HISTORY QUESTIONS** |  |
| 1. **Have you previously been diagnosed with a hereditary cancer syndrome either through a positive genetic test or clinical history? For examples: hereditary breast and ovarian cancer syndrome (BRCA 1/2, Lynch syndrome (EPCAM, MLH1, MSH2, MSH6, MLH1, PMS2), Li- Fraumeni (TP53), familial adenomatous polyposis (APC), Muir-Torre syndrome, Peutz-Jeghers syndrome (STK11), familial atypical multiple mole melanoma syndrome (FAMMM) (CDKN2A), or other hereditary cancer risk**    Yes, I have had genetic testing  Yes, but I have not yet had genetic testing   No  Unknown  I have a family member that has been diagnosed with a hereditary cancer syndrome | 3 3 0 0 3 |
| 1. **Have you ever had one of the following cancers?**  Breast  Colon    Malignant melanoma  Ovarian  Prostate  Uterine  Pancreatic  None of the above | 1 1 1 3  1 1 3 0 |
| 1. **Were you diagnosed with any of the above cancers before or at the age of 50?**  Yes  No | 2 0 |
| 1. **What is your biological mother’s cancer history?**    Pancreatic  Breast   Ovarian  Uterine  Colon   Melanoma  Other cancer  None of the above | 3 1 3 1 1 1 0 0 |
| 1. **Was your mother diagnosed with any of the above cancers before or at the age of 50?**  Yes  No  Unsure | 2 0 0 |
| 1. **What is your biological father’s cancer history?**    Pancreatic  Breast   Prostate  Colon   Melanoma  Other cancer  None of the above | 3 3 1 1 1 0 0 |
| 1. **Was your father diagnosed with any of the above cancers before or at the age of 50?**    Yes  No  Unsure | 2 0 0 |
| 1. **Do either of your biological parents have any Ashkenazi (Eastern European) Jewish ancestors that you are aware of?**   **** Yes, my mother   Yes, my father   Yes, both my biological parents   I do not know my biological parents  **** No  Unknown | 1 1 2 0 0 0 |
| 1. **Do you have any biological siblings who have been diagnosed with the following cancers?**    Pancreatic   Breast   Prostate  Ovarian   Colon   Melanoma   Uterine   I have no siblings  None of the above  I have no siblings | 3 1 1 3 1 1 1 1 0 0 0 |
| 1. **Were any of your siblings diagnosed with any of the above cancers before or at the age of 50?**    Yes  No  Unknown | 2 0 0 |
| 1. **Do you have any biological children who have been diagnosed with cancer? (Please select all that apply)**    Pancreatic   Breast   Prostate  Ovarian   Colon   Melanoma   Uterine   Other cancer  None of the above  I have no children | 3 1 1 3 1 1 1 0 0 0 |
| 1. **Were any of your children diagnosed with any of the above cancers before or at the age of 50?**    Yes  No  Unknown | 2 0 0 |
| 1. **Do you have any second-degree relatives (aunt/uncle, niece/nephew, grandparent) who have been diagnosed with the following cancer(s) or other diseases? (Please select all that apply)**  Pancreatic    Breast   Prostate  Ovarian   Colon   Melanoma   Uterine   None of the above  Other | 3 1  1 3 1 1 1 0 0 |
| 1. **Were any of your second-degree relatives diagnosed with any of the above cancers before or at the age of 50?**    Yes  No  Unknown | 2 0 0 |

**Supplemental table 2.** Details of five individuals who underwent previous genetic testing in which a pathogenic variant was identified

| **Characteristics, reason of visit** | **Gene** | **Risk score** |
| --- | --- | --- |
| 60/F, Genetic susceptibility | *BRCA1* and *BRCA2* | 11 |
| 82/M, Recurrence of renal cell carcinoma in gastric wall | *Prothrombin G20210A* (non-cancer associated) | 4 |
| 32/M, Evaluation recurrent pancreatitis | *COL6A1* (non-cancer associated) | 3 |
| 52/F, Candida esophagitis | *CHEK2* | 3 |
| 61/F, Surveillance for endocrine neoplasia | *MEN1* | 2 |
